# A molecular structure matching approach to efficient identification of endogenous mammalian biochemical structures

**DOI:** 10.1186/1471-2105-16-S5-S11

**Published:** 2015-03-18

**Authors:** Mai A Hamdalla, Reda A Ammar, Sanguthevar Rajasekaran

**Affiliations:** 1 Computer Science and Engineering Department, University of Connecticut, Connecticut, USA; 2 Computer Science and Information Systems Department, Helwan University, Cairo, Egypt

**Keywords:** Metabolomics, Metabolite Identification, Chemical Structure Matching, Biochemical Structures

## Abstract

Metabolomics is the study of small molecules, called metabolites, of a cell, tissue or organism. It is of particular interest as endogenous metabolites represent the phenotype resulting from gene expression. A major challenge in metabolomics research is the structural identification of unknown biochemical compounds in complex biofluids. In this paper we present an efficient cheminformatics tool, BioSMXpress that uses known endogenous mammalian biochemicals and graph matching methods to identify endogenous mammalian biochemical structures in chemical structure space. The results of a comprehensive set of empirical experiments suggest that BioSMXpress identifies endogenous mammalian biochemical structures with high accuracy. BioSM*Xpress *is 8 times faster than our previous work BioSM without compromising the accuracy of the predictions made. BioSM*Xpress *is freely available at http://engr.uconn.edu/~rajasek/BioSMXpress.zip

## Introduction

Metabolomics is the comprehensive, qualitative, and quantitative study of all the small molecules, called metabolites, in an organism [1]. A major challenge in metabolomics is the interpretation of the vast amount of data produced by the high-throughput techniques used for information extraction and data interpretation [2,3]. The existence of several on-line chemical structure databases has provided a vital support for molecular identification by allowing the search for candidate compounds using experimentally determined features with computationally simulated features. Such searches often result in a large number of false positives, making identification of the compound under investigation extremely difficult. Hence, cheminformatics methods are needed to efficiently search such large chemical databases and potentially identify unknown endogenous biochemical compounds. Several methods have been developed with the objective of discriminating between candidate structures that are synthetic and those that are biochemical. The first attempt to solve this problem was reported by Nobeli *et al*. [4] who used two-dimensional molecular structures to manually derive a library of 57 structural fragments commonly found in 745 E. coli metabolites. Such fragments were used to reveal the main constituents of metabolites and to assist in the classification of the metabolome into biochemically relevant classes. In related work, Gupta and Aires-de-Sousa [5] and Peironcely *et al*. [6] employed fingerprints and random forest classifiers [7] to classify endogenous biochemical compounds. Molecular fingerprints represent the structure of a molecule as a list of binary values (0 or 1) that indicate the presence or absence of structural features in the molecule [8]. Gupta and Aires-de-Sousa's model correctly annotates 95% of the 1,811 compounds downloaded from the Kyoto Encyclopedia of Genes and Genomes (KEGG) [9] used for training their model. While Peironcely *et al*. reported that 96% of 457 compounds downloaded from the Human Metabolome Database (HMDB) [10], not used for training the model, were classified as endogenous. In our previous work [11], we developed BioSM, a molecular classifier that can identify endogenous mammalian biochemical structures contained within chemical structure space. BioSM uses the structures of known endogenous mammalian biochemical compounds to aid in the classification process, as opposed to other works that use fragments of known structures. BioSM correctly predicted 95% of 1,388 (KEGG) compounds as endogenous mammalian biochemical in a set of leave-one-out cross validation experiment. Additionally, 89% of 2,330 compounds downloaded from HMDB were identified as endogenous metabolites. One of BioSM's limitations, granting its encouraging results, was its need to exhaustively search all known biochemical structures to be able to make a decision about the candidate compound under investigation which resulted in an undesirably high run time. In this paper we propose an efficient cheminformatics tool, which can be used to identify biological compounds based on their molecular structures, called BioSMXpress. Similar to our previous work, the prediction method applied by BioSMXpress relies on a set of endogenous mammalian biochemical compounds obtained from the KEGG database, hereafter referred to as scaffolds. In a nutshell, BioSMXpress is designed to predict that a given query structure is biological after encountering **exactly one **scaffold match satisfying a given threshold. This threshold is based on the number of atoms in both the query structure and the scaffold being examined. Knowing this gives us the opportunity to avoid the need to exhaustively search the entire scaffolds list before making a decision about the query compound with confidence. To do this efficiently, BioSMXpress selects the scaffolds that have the potential to promote the candidate in the least possible time. Then, only those scaffolds, with enough atoms to satisfy the given threshold, are checked against the candidate compound for similarity.

## Materials and methods

BioSM*Xpress *was designed as an enhancement to BioSM with the aim of making the least possible number of structure comparisons to efficiently identify biochemical structures with the aid of a scaffolds list. BioSM*Xpress *decides if a candidate structure is biochemical based upon how similar that structure is to any of the structures in the scaffolds list. In this work, two molecular structures are considered to be a "match", if the smaller structure is an exact substructure (atom and bond types) of the larger structure being compared. The underlying cheminformatics functionality of BioSM*Xpress *is based on an open source Java based toolkit called the Small Molecule Subgraph Detector (SMSD) [12]. This toolkit is used to find the maximum common sub-graph between small molecules using atom type matches and bond sensitivity information. In addition to SMSD, BioSM*Xpress *uses Marvin, a chemical structure processing software, to generate both the canonical SMILES and atom counts from structure data files (.sdf) for all the compounds described in this work.

### Computational algorithm

Here, we introduce a tool that can efficiently identify small endogenous mammalian biochemical structures from chemical structure space. First we will start by defining some notations followed by a detailed explanation of the computational model behind BioSM*Xpress*. Let *c_q _*be the molecular structure of a query compound, *S *= {*s*_1_, *s*_2_,...,*s_n_*} be a set of *n *small biological compounds (scaffolds). Let *s_x _*∼ *c_q _*indicate that scaffold *s_x _*is a substructure of candidate compound *c_q_*, and *AC *(*s_x_*) represent the number of atoms in compound *s_x_*. Let *minAC *define the minimum number of atoms required in a scaffold *s_y _*to identify *c_q _*as biological if *c_q _*∼ *s_y_*. If two molecular structures *r *and *q *were found to be a match, a similarity score

(1) $$Sc=\frac{AC\left(r\right)}{AC\left(q\right)}$$

is computed where *r *∼ *q*. Based on a given substructure threshold (*subThr*), the minimum atom count is computed as

(2) $$minAC=\left[AC\left(c_{q}\right)*subThr\right].$$

Similarly, based on a given superstructure threshold (*superThr *), BioSM*Xpress *computes the maximum atom count,

(3) $$maxAC=\left[\frac{AC\left(c_{q}\right)}{\text{sup}erThr}\right].$$

Finally, let $\bar{S}=\left\{s_{1}^{\prime },s_{2}^{\prime },\ldots ,s_{l}^{\prime }\right\}|l\leq n$ be the scaffold list assigned to *c_q _*where $\bar{S}\subseteq S$. A scaffold $s_{x}^{\prime }\in S$ is assigned to $\bar{S}$ if and only if $minAC\leq AC\left(s_{x}^{\prime }\right)\leq maxAC$. Please note that each candidate structure with a different atom count is provided with a different set of scaffolds in $\bar{S}$. Once $\bar{S}$ is populated with the appropriate scaffolds for *c_q_*, BioSM*Xpress *examines *c_q _*against each of those scaffolds. As soon as a match (substructure or superstructure) is found, BioSM*Xpress *predicts that *c_q _*is biological and terminates. Otherwise, it's predicted to be nonbiological. Values for *subThr *and *superThr *are determined by cross validation as described in the following section.

**Figure 1:**
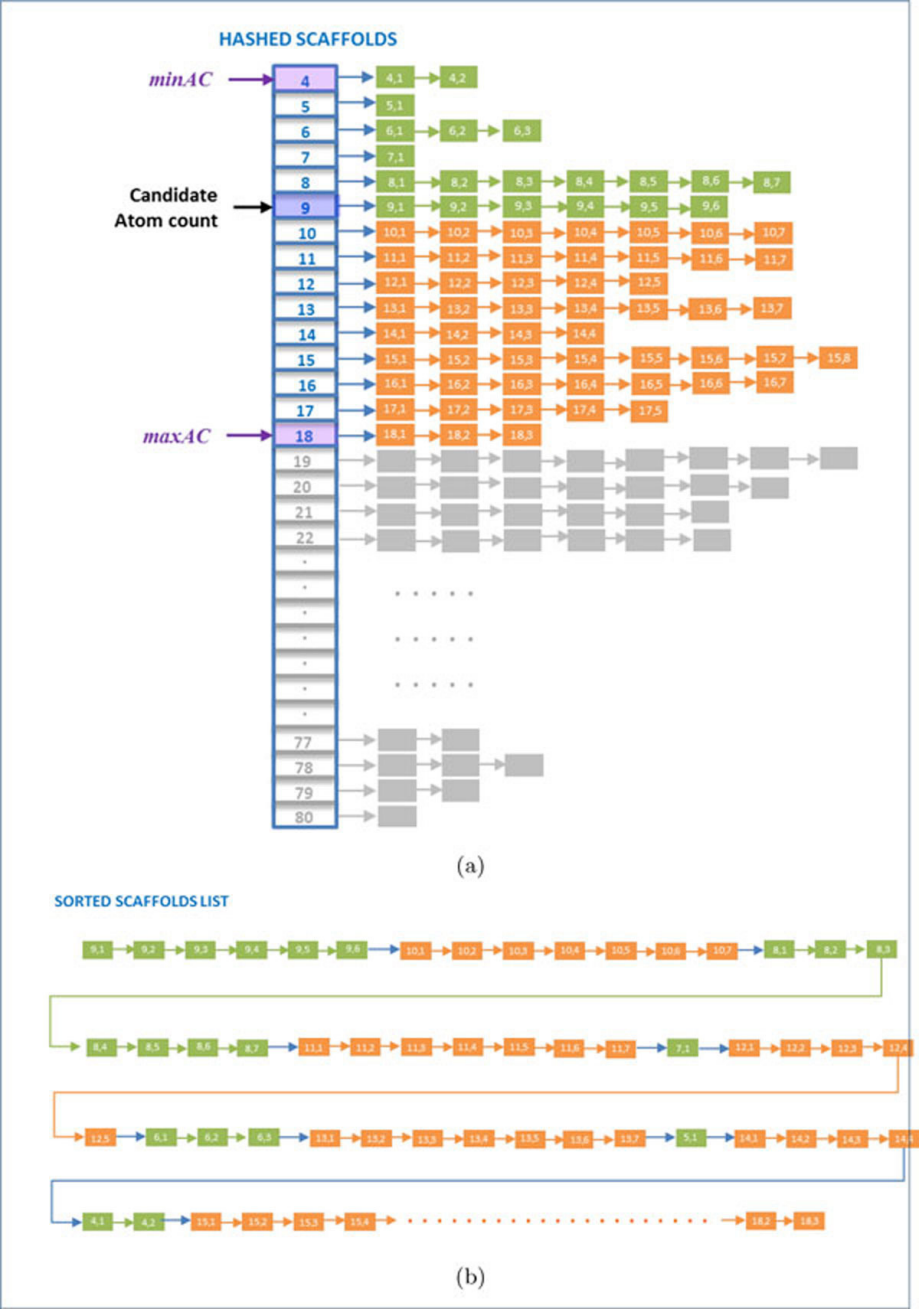
**Scaffold selection and sorting process**. In this example, it is assumed that the candidate compound (*c_q_*) consists of 9 atoms and that *subThr *= 0.5 and *superThr *= 0.51. Therefore, *minAC *= [9 ∗ 0.5] = 4 and $maxAC=\;\left\lceil \frac{9}{0.51}\right\rceil =18$. (a) The hashed scaffolds list with *minAC *and *maxAC *identified. (b) The sorted scaffolds list consists of all the scaffolds with 9 atoms followed by those with 10 atoms followed by those with 8 atoms and so on.

In addition to that, BioSM*Xpress *orders the potential scaffolds in $\bar{S}$ such that scaffolds with atom counts closer to the candidate atom count are examined first followed by those with a larger atom count difference. In this case, once a match is found the search terminates and it is guaranteed that this is the best possible match (as a substructure or superstructure). Figure 1 illustrates a visual example of the BioSM*Xpress *scaffolds ordering process.

### Datasets

We will briefly describe the source and nature of the datasets selected to train and validate BioSM*Xpress*. Since these datasets will be used to compare between BioSM*Xpress *and BioSM in terms of prediction accuracy, we utilized the same datasets and followed the same curation steps in [11]. In each dataset, compounds with any of the following characteristics were eliminated: (1) compounds with elements other than C, H, N, O, P and S; (2) compounds with less than 4 atoms and more than 53 atoms (explained below); (3) compounds that were polymers; (4) charged structures except those in which the charge was due to quaternary amines or sulfonium ions; (5) compounds with duplicate structures; and (6) compounds with disjoint structures. We start by defining compounds used to define biological versus non-biological in chemical structure space in this study.

#### Biological Dataset (Scaffolds list)

The KEGG database was chosen as the source of endogenous mammalian compounds. The list of 1,564 mammalian scaffolds (KEGGscafs) defined in [11] were used to represent the biochemical structure space in BioSM*Xpress*. Each compound in the scaffolds list comprises of a number of atoms from 4 to 80 atoms per compound.

#### Non-Biological Dataset (Synthetic compounds list)

The Chembridge http://www.chembridge.com and Chemsynthesis http://www.chemsynthesis.com databases served as the sources of compounds representing the non-biological chemical space. These databases were selected because they comprise synthetic compounds for chemical synthesis and drug screening and design. After curation, a set of 375,930 structures represented the synthetic compounds list. Chemsynthesis and Chembridge databases mainly contain compounds with low molecular weights (a maximum atom count of 53 atoms per compound). Accordingly, 143 of the 1,564 KEGGscafs (with atom count between 54 and 80) were eliminated from any testing set throughout this study and were only used for superstructure scaffold matching. This restriction was enforced to ensure that the sole discrimination between a compound being biological or non-biological is based on the structure of a compound and not on the number of atoms in that compound.

#### Training Dataset

A total of 2,842 compounds, with at least 4 atoms and at most 53, were used to train and test our predictive model. Half of those compounds were obtained from the scaffolds list (representing the endogenous mammalian chemical space) and the other half from the synthetic compounds list (representing the non-biological chemical space). The later molecules were randomly selected from the synthetic dataset to match the atom count distribution of the 1,421 biological set.

#### Independent Datasets

To estimate the performance of our predictive model and compare it with that of BioSM, four external validation sets were used: one set of putative human metabolites, one set of plant secondary metabolites, one set of drugs, and one set of synthetic compounds. For each dataset, any compound with a structure identical to any of those in the scaffolds list was removed. Also, structures found in more than one dataset were removed from all datasets except one. Molecules in each dataset had to satisfy both mass (50 - 700 Da) and atom count (4 - 53 atoms) constraints to allow for a fair comparison between BioSM*Xpress *and BioSM. Additionally, compounds with at least one non-biological substructure (NBS) were eliminated. NBSs are substructures that are not commonly found in mammalian biochemical compounds. This decision was based on our interest in comparing the core predictive models of BioSM and BioSM*Xpress *since in reality, NBS filters will be applied to both models before any scaffold comparisons are involved. For more details on the curation process followed please refer to [11].

The following is a brief description of the five datasets. Please note that the numbers of compounds reported below refer to the datasets after curation. The first dataset consisted of 2,329 compounds and was obtained from HMDB version 2.5 representing putative human metabolites. The second dataset consists of 2,416 secondary plant metabolites, as specified by KEGG, representing plant structures. The drug dataset was represented by 3,282 compounds and was obtained from DrugBank [13] version 3.0 and from the 1989 USAN and the USP Dictionary of Drug Names. A randomly chosen set of approximately 46,203 molecules was from the National Center for Biotechnology Information's (NCBI) PubChem database [14]. PubChem is the largest freely accessible compound database currently available. Finally, a set of 374,509 compounds from the Chembridge and Chemsynthesis databases, not used in training the model, were used as a synthetic compound test set.

## Results and discussion

### Classification methods selection

Four classification methods were proposed for BioSM*Xpress *specifically, the SSF method which refers to finding a substructure scaffold match or a superstructure scaffold match in the sorted scaffolds list was utilized. SSSF refers to searching for the best substructure scaffold similarity score (*Sc_sub_*), if existent, and the best superstructure scaffold similarity score (*Sc_super_*), if existent. It declares the candidate as biological if *Sc_sub _*+ *Sc_super _*≥ *sumThr*. From my experience with BioSM, I found that distributing candidates into mass bins, with each bin having its own threshold values, showed an improvement in the prediction quality. Thus, we decided to test if the same concept applies here. I split the set of test compounds into five bins based on the number of atoms per compound and used CV to evaluate the model. This introduced two more methods, SSB and SSSB, similar to SSF and SSSF respectively, except that there are independent thresholds assigned to each bin.

Cross Validation (CV) is a method used for estimating how accurately a predictive algorithm will perform in practice while avoiding overfitting as well as tuning meta-parameters [15]. In this study, we used a nested CV framework where parameter tuning was performed by executing 5-fold CV on the training data and the classification accuracy was empirically assessed using 2-fold CV on the testing data. For the results of each training fold, the score where SENS = SPEC was recorded as the cutoff threshold of that fold. This process was repeated 5 times to insure that each of the 5 parts was evaluated. Then the average thresholds of all five training sets were used as the cutoff values when evaluating the CV testing data as explained in.

We ran 15 CV experiments to evaluate the performance of each method. Several accuracy measures were applied to each experiment such as sensitivity $\left(SENS=\frac{TP}{TP+FN}\right)$, specificity $\left(SPEC=\frac{TN}{TN+FP}\right)$, and matthews correlation coefficient $\left(MCC=\frac{TP.TN-FP.FN}{\sqrt{\left(TN+FN\right).\left(TN+FP\right).\left(TP+FN\right).\left(TP+FP\right)}}\right)$. Were *TP *refers to the number of correctly identified compounds while *FP *refers to the number of incorrectly identified molecules. Similarly, *TN *refers to the number of correctly rejected compounds and *FN *refers to the number of those incorrectly rejected. The mean and standard deviation of the 15 experiments are displayed in Table 1. The highest sensitivity of 90% was obtained by the SSF classifier. At the same time, SSF suffered from the lowest specificity of 55% only. Another observation is that the application of *sumThr *improved the specificity significantly, 82% (SSSB) and 71% (SSSF) versus 62% (SSB) and 55% (SSF) but affected the sensitivity negatively (53% and 73%, respectively). SSB had the best MCC of 51% and hence was selected as the method of choice for BioSM*Xpress *with a sensitivity of 86% and a specificity of 62%.

**Table 1:** Mean and standard deviation of accuracy measures obtained for 15 cross validation experiments using 4 different scoring.

		SSF	SSSF	SSB	SSSB
**SENS**	**Mean**	0.90	0.73	0.86	0.58
	
	**StdDev**	0.02	0.04	0.02	0.03

**SPEC**	**Mean**	0.55	0.71	0.62	0.82
	
	**StdDev**	0.04	0.05	0.04	0.03

**MCC**	**Mean**	0.41	0.45	0.51	0.48
	
	**StdDev**	0.03	0.02	0.05	0.05

### Leave-One-Out cross validation analysis

As an additional method to evaluate how well BioSM*Xpress *can identify endogenous mammalian biochemical structures using the SSB classifier, we carried out a set of LOOCV experiments using the SSB method with the averaged *subThr*, *superThr *and bin boundaries determined by CV as explained in the Methods section. Here, 1,421 experiments representing KEGG structures (with atom count between 4 and 53 atoms/compound) were carried out. Please note that in every experiment, the scaffolds list was composed of 1,420 compounds plus 143 compounds (those with atom count between 54 and 80 atoms/compound) as the scaffolds list. As a result, BioSM*Xpress *annotated 94% of the 1,421 compounds as being biological structures.

**Figure 2:**
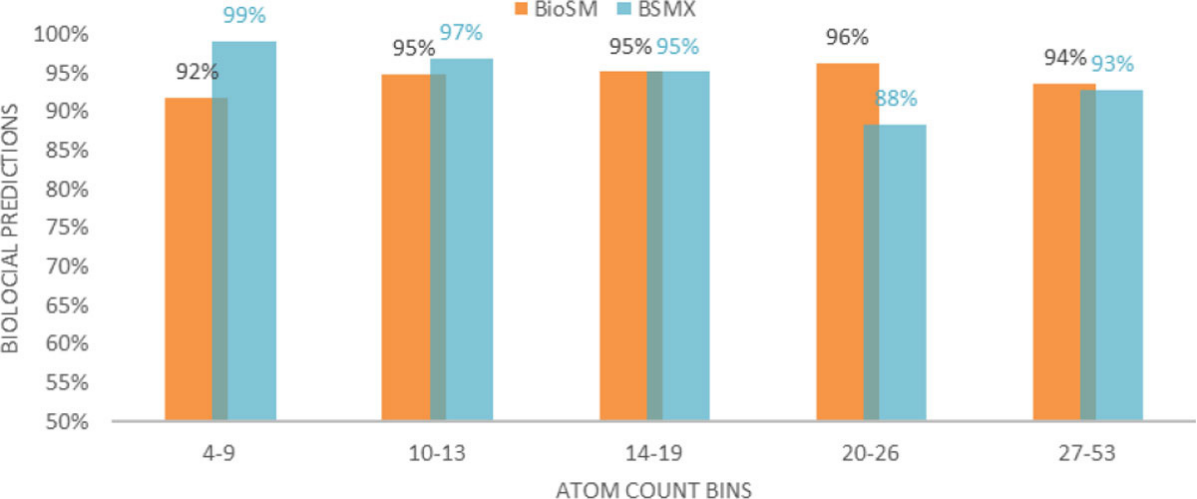
**Biological predictions resulting from a set of LOOCV experiments by BioSM*Xpress *and BioSM with 1,387 KEGG compounds**. Compounds were binned by atom count.

Using 1,387 scaffolds in a set of LOOCV experiments implemented by BioSM*Xpress *and BioSM independently were implemented and compared. These 1,387 compounds were the scaffolds that satisfied the constraints of both BioSM and BioSM*Xpress *(mass in the range of 50 - 700 Da and number of atoms in the range of 4 - 53). BioSM was capable of identifying 94.5% of the 1,387 scaffolds as biochemical structures while BioSM*Xpress *identified 94.2%. Figure 2 shows the breakdown of the results of this comparison with compounds binned by atom count.

BioSM*Xpress *performed best when identifying compounds in the first bin (99% positive identification of biochemical compounds) while BioSM was able to predict only 92% of those compounds. In general, Figure 2 indicates that BioSM*Xpress *is better at identifying biochemical compounds in the first and second bins (99% and 97%, respectively), while BioSM is better at identifying biochemical compounds in the fourth and fifth bins (96% and 94%, respectively).

A note worth mentioning is that in addition to the 1,387 scaffolds annotated, BioSM*Xpress *was able to positively identify 34 compounds in the scaffolds list that were rejected by BioSM without classification due to mass restrictions (masses were greater than 700 Daltons). This indicates that BioSM*Xpress *has broadened the range of compounds examined just by restricting the number of atoms in a candidate compound versus its molecular mass.

### Prospective validation

Independent datasets containing plant secondary metabolites, drugs, human metabolites, synthetic molecules, and PubChem compounds were classified by BioSM*Xpress*. Subsequently, the same datasets were also annotated by BioSM. Table 2 presents a comparison of BioSM's predictions versus those of BioSM*Xpress*. The results indicate that 91% of the 2,329 HMDB molecules were correctly classified as endogenous mammalian metabolites while 88% of them were identified by BioSM. Predictions for the 2,416 plant metabolites by *BioSMXpress *and BioSM were comparable with 72% and 73%, respectively. As for the 3,282 drug compounds, 58% were predicted to be biological by BioSM*Xpress *versus 62% by BioSM. In contrast, only 25% of the randomly selected 46,203 PubChem compounds were predicted as biological by BioSM*Xpress *as opposed to 35% by BioSM.

**Table 2:** Predictive results using the SSB classifier for 6 different datasets.

Dataset	Number of Compounds	BioSM	BioSMXpress
**HMDB Compounds**	2,329	88%	91%

**Plant Metabolites**	2,416	73%	72%

**Drug Compounds**	3,282	62%	58%

**PubChem Compounds**	46,203	35%	25%

**Synthetic Compounds**	374,509	33%	36%

In addition to these four prospective datasets, a set of 374,509 synthetic compounds (represented by Chembridge and Chemsynthesis compounds) were evaluated by BioSM*Xpress *and BioSM with 36% and 33% of these being predicted to be biochemical, respectively. Overall, the comparison in Table 2 shows that the biochemical prediction percentages made by BioSM and BioSM*Xpress *are practically comparable except that BioSM*Xpress *predicted 10% lesser compounds of the PubChem compounds as biological.

### Execution and CPU time comparison

**Figure 3:**
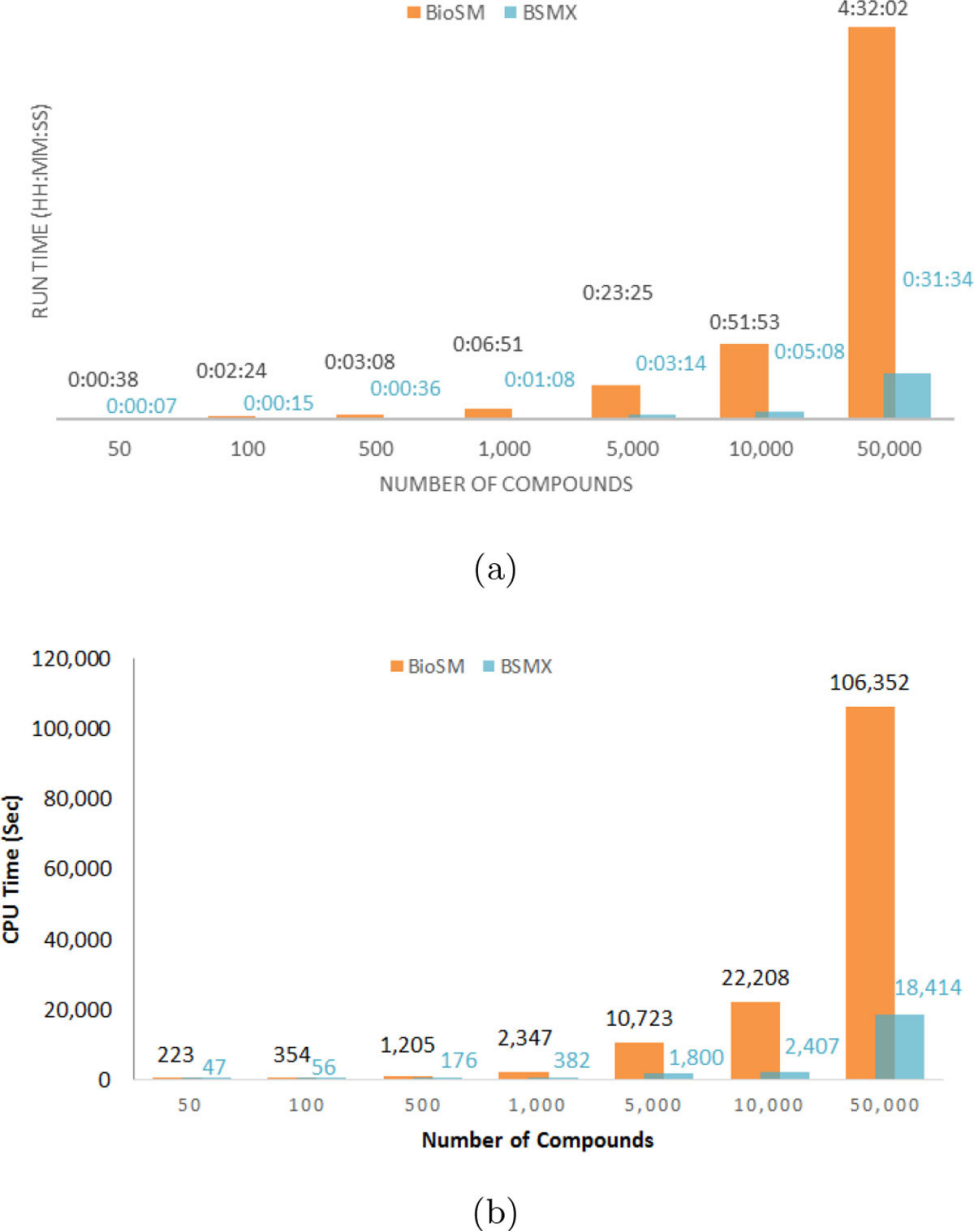
**(a) Average runtime (in hh:mm:ss) needed to make predictions using BioSM versus BioSM*Xpress***. (b) Average CPU time (in seconds) for BioSM and BioSM*Xpress *when annotating sets of compounds of different sizes.

Now that we have shown that the predictive performance of BioSM*Xpress *is analogous to BioSM, in this section we discuss their time performance. We used a high-end cluster http://becat.uconn.edu/hpc/ hosted by the School of Engineering and the Taylor L. Booth Engineering Center for Advanced Technology (BECAT) at the University of Connecticut to run and compare the performance of both BioSM and BioSM*Xpress*. We ran both classifiers with a set of randomly generated datasets as candidate datasets for prediction. Each dataset was evaluated by both predictive models under the same circumstances (same number of cluster nodes, threads, same scaffolds list, etc.) and the time for each model was recorded. We were also interested in comparing the CPU time utilized by both BioSM and BioSM*Xpress*. CPU time is the amount of time for which a central processing unit (CPU) was used for processing instructions of a computer program or operating system, as opposed to, for example, waiting for input/output operations. Figure 3 shows the average CPU time utilized by each of the classifiers when making predictions for each data set size (50 - 50,000 compounds). Similar to response time, BioSM*Xpress *has outperformed BioSM by utilizing an average of 7 times less CPU time. We generated multiple candidate datasets with 50, 100, 500, 1,000, 5,000, 10,000, and 50,000 compounds. Each dataset was composed of randomly selected compounds from a pool of all the independent datasets used in this study as described in the Methods section. To ensure that the only factor we are measuring is the number of compounds in a set no matter what the nature of the compounds included is, we generated multiple random datasets (specifically 3) with the same number of compounds for each size required. So we ran 3 groups each containing 50 randomly selected compounds, 3 groups of 100 compounds and so on, and then reported the average response time of each group size. Figure 3 displays the average run time of BioSM versus that of BioSM*Xpress *when annotating datasets of sizes 50 to 50,000 compounds as explained above.

Obviously, BioSM*Xpress *impressively outperformed BioSM across all datasets. BioSM*Xpress *was 10 times faster than BioSM when analyzing 10,000 compounds. Across all datasets examined, BioSM*Xpress *provided an 8 times average speed up over BioSM. Another interesting observation is that it takes BioSM an average of 6 minutes and 51 seconds to evaluate 1,000 compounds while it takes BioSM*Xpress *an average of 5 minutes and 8 seconds to evaluate 10,000 compounds (10 times more compounds in less time).

This drastic difference in run time and CPU time can be explained by observing the number of scaffold comparisons required by each predictive model to make a prediction about any given candidate compound. BioSM needs to compare the candidate structure with each and every structure in the scaffolds list accumulating scores and then finally comparing that score with a threshold to make a prediction. BioSM*Xpress *intelligently selects and sorts the scaffolds that would produce the highest match scores, based on the thresholds, if they were to match the candidate. Only a portion of the scaffolds are added to the list that is actually used by BioSM*Xpress *as the scaffolds list and once a match is found the candidate is predicted to be biological with no other computational steps further needed.

## Conclusions

In this paper, we describe the development and validation of BioSM*Xpress*, an efficient supervised cheminformatics tool that uses endogenous mammalian biochemical scaffolds to predict whether a candidate chemical structure is biochemical or synthetic. BioSM*Xpress *is at average 8 times faster than BioSM without compromising the accuracy of the predictions. BioSM*Xpress *was able to correctly classify 94% of 1,421 biochemical compounds in a set of leave-one-out cross validation experiment. Thus BioSM*Xpress *may be useful for searching large chemical databases in metabolomics applications where the number candidates is extremely large as well as the number of potential false positives in an efficient manner.

## Competing interests

The authors declare that they have no competing interests.

## Authors' contributions

M.A.H. was responsible for designing the algorithm, software development, and manuscript preparation. She was also responsible for testing and benchmarking BioSM*Xpress*. S.R. was involved in the overall supervision of the project, manuscript preparation, intellectual input, and guidance. All authors have given approval to the final version of the manuscript.

## Declarations

This work has been supported in part by the NIH Grant R01-LM010101 and the Booth Engineering Center for Advance Technology (BECAT) at the University of Connecticut.

This article has been published as part of *BMC Bioinformatics *Volume 16 Supplement 5, 2015: Selected articles from the 10th International Symposium on Bioinformatics Research and Applications (ISBRA-14): Bioinformatics. The full contents of the supplement are available online at http://www.biomedcentral.com/bmcbioinformatics/supplements/16/S5.

## References

[B1] Villas-Bôas SG Bruheim P The potential of metabolomics tools in bioremediation studies Omics : a journal of integrative biology 2007 11 3 305 13 doi:10.1089/omi.2007.0005 10.1089/omi.2007.0005 17883341

[B2] Kertesz T Hill DW Albaugh D Hall L Hall L Grant DF Database searching for structural identification of metabolites in complex biofluids for mass spectrometry-based metabonomics Bioanalysis 2009 1 9 1627 1643 10.4155/bio.09.145 21083108

[B3] Roessner U Bowne J What is metabolomics all about? BioTechniques 2009 46 5 363 365 doi:10.2144/000113133 10.2144/000113133 19480633

[B4] Nobeli I Ponstingl H Krissinel EB Thornton JM A structure-based anatomy of the E.coli metabolome Journal of Molecular Biology 2003 334 4 697 719 10.1016/j.jmb.2003.10.008 14636597

[B5] Gupta S Aires-de-Sousa Ja Comparing the chemical spaces of metabolites and available chemicals: models of metabolite-likeness Molecular Diversity 2007 11 1 23 36 doi:10.1007/s11030-006-9054-0 10.1007/s11030-006-9054-0 17447158

[B6] Peironcely JE Reijmers T Coulier L Bender A Hankemeier T Understanding and classifying metabolite space and metabolite-likeness PloS One 2011 6 12 doi:10.1371/journal.pone.0028966 10.1371/journal.pone.0028966 PMC3237584 22194963

[B7] Breiman L Random forests Machine Learning 2001 45 Kluwer Academic Publishers 5 32 http://onlinelibrary.wiley.com/doi/10.1002/cbdv.200490137/abstract 10.1023/A:1010933404324

[B8] James CA Weininger D Delany J Irvine, CA and Santa Fe, NM Fingerprints - Screening and Similarity Daylight Theory Manual 2000 Daylight Chemical Information Systems, Inc http://www.ncbi.nlm.nih.gov/pubmed/23055325 doi:10.1002/mus.23684.

[B9] Kanehisa M Goto S Kawashima S Nakaya A The KEGG databases at GenomeNet Nucleic Acids Research 2002 30 1 42 46 10.1093/nar/30.1.42 11752249 PMC99091

[B10] Wishart DS Knox C Guo AC Eisner R Young N Gautam B Hau DD Psychogios N Dong E Bouatra S Mandal R Sinelnikov I Xia J Jia L Cruz Ja Lim E Sobsey Ca Shrivastava S Huang P Liu P Fang L Peng J Fradette R Cheng D Tzur D Clements M Lewis A De Souza A Zuniga A Dawe M Xiong Y Clive D Greiner R Nazyrova A Shaykhutdinov R Li L Vogel HJ Forsythe I HMDB: a knowledgebase for the human metabolome Nucleic Acids Research 2009 37 Database 603 610 doi:10.1093/nar/gkn810 10.1093/nar/gkn810 PMC2686599 18953024

[B11] Hamdalla MA Mandoiu II Hill DW Rajasekaran S Grant DF BioSM: A chemoinformatics tool for identifying biochemical structures in chemical structure space Journal of Chemical Information and Modeling 2012 10.1021/ci300512q PMC3866231 23330685

[B12] Rahman SA Bashton M Holliday GL Schrader R Thornton JM Small Molecule Subgraph Detector (SMSD) toolkit Journal of Cheminformatics 2009 1 12 doi:10.1186/1758-2946-1-12 10.1186/1758-2946-1-12 PMC2820491 20298518

[B13] Knox C Law V Jewison T Liu P Ly S Frolkis A Pon A Banco K Mak C Neveu V Djoumbou Y Eisner R Guo AC Wishart DS DrugBank 3.0: a comprehensive resource for 'omics' research on drugs Nucleic Acids Research 2011 39 Database 1035 41 doi:10.1093/nar/gkq1126 10.1093/nar/gkq1126 PMC3013709 21059682

[B14] Bolton EE Wang Y Thiessen PA Bryant SH PubChem: Integrated Platform of Small Molecules and Biological Activities Annual Reports in Computational Chemistry 2008 4 4 Chap 12 American Chemical Society, Washington 217 241

[B15] Hastie T Tibshirani R Friedman J The Elements of Statistical Learning 2009 2 Springer http://www-stat.stanford.edu/texttildelowtibs/ElemStatLearn/

